# Retraction of: “LncRNA CASC15 inhibition relieves renal fibrosis in diabetic nephropathy through downregulating SP-A by sponging to miR-424”

**DOI:** 10.1515/med-2025-9991

**Published:** 2025-10-24

**Authors:** Hui Li, Jian Hao, Weimin Yu

**Affiliations:** Department of Nephrology, Shanxi Bethune Hospital, Shanxi Academy of Medical Sciences, Tongji Shanxi Hospital, Third Hospital of Shanxi Medical University, Taiyuan, 030032, China; Department of Nephrology, Shanxi Bethune Hospital, Shanxi Academy of Medical Sciences, Tongji Shanxi Hospital, Third Hospital of Shanxi Medical University, No. 99 Longcheng Street, Xiaodian District,, Taiyuan, 030032, China

Retraction of: Li H, Hao J, Yu W. LncRNA CASC15 inhibition relieves renal fibrosis in diabetic nephropathy through downregulating SP-A by sponging to miR-424. Open Medicine 2023; 18(1): 20230710, DOI: 10.1515/med-2023-0710.

The retraction has been agreed upon at the request of the corresponding author, Dr. Jian Hao, with the consent of all co-authors, the Editor-in-Chief, Professor Vittorio Calabrese, and the publisher, Walter de Gruyter GmbH. The retraction is due to plagiarism of the plots included in Figures 4b and 8b, which were copied from multiple sources, indicating systematic publication manipulation. In addition, the authors acknowledged the undisclosed outsourcing of the research, which undermines the legitimacy of the study.

